# Traumatic inguinal hernia after fall from truck on a broom

**DOI:** 10.1016/j.tcr.2022.100617

**Published:** 2022-01-26

**Authors:** Daniel Bakker, Louis de Jong, Jesse van Buijtenen, Maria Verseveld

**Affiliations:** a Erasmus University Rotterdam, Faculty of Medicine, Erasmus University Medical Center, Wytemaweg 80, 3015 CN Rotterdam, the Netherlands; b Franciscus Hospital, Department of General Surgery, Kleiweg 500, 3045 PM Rotterdam, the Netherlands

**Keywords:** Traumatic hernia, Inguinal canal, Abdominal wall, Case report, Laparoscopy

## Abstract

**Background:**

Inguinal hernias are among the most common abdominal wall hernias but rarely caused by penetrating trauma.

**Case presentation:**

We report a case of a 61-year-old patient with a traumatic inguinal hernia after penetrating injury through the inguinal canal. Local inspection of the intestines and abdominal cavity showed no fecal spill, blood clots or signs of contamination. Therefore, no laparoscopy or laparotomy was initiated. The abdominal wall was closed using a mesh patch. No infections or re-herniation occurred.

**Conclusion:**

Clinicians could consider local exploration in the treatment of traumatic inguinal hernias.

## Case-presentation

**Fig. 1:**
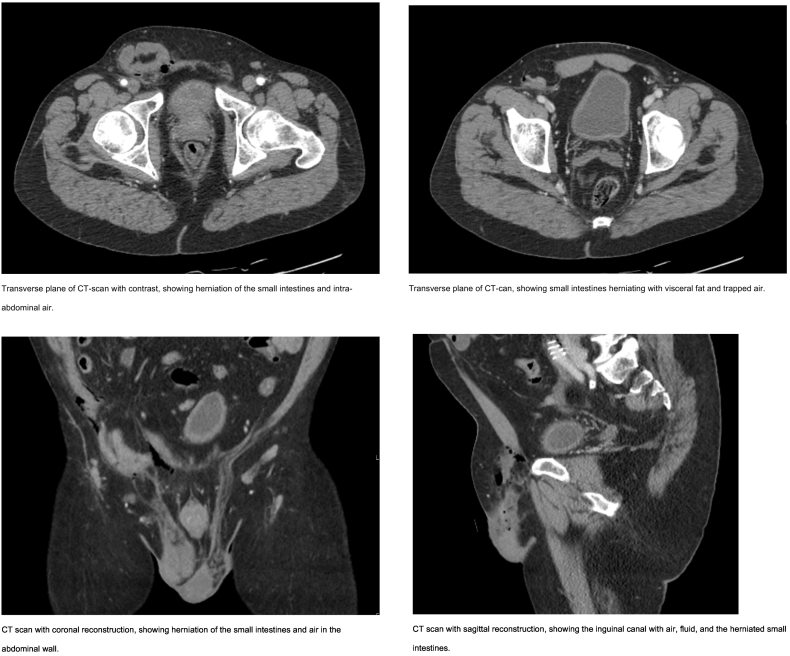
Patients CT images. Transverse plane of CT-scan with contrast, showing herniation of the small intestines and intra abdominal air. Transverse plane of CT-scan with contrast, showing herniation of the small intestines with visceral fat and trapped air. CT scan with coronal reconstruction, showing herniation of the small intestines and air in the abdominal wall. CT scan with sagittal reconstruction, showing the inguinal canal with air, fluid, and the herniated small intestine.

A 61-year old man was admitted to the emergency department with severe abdominal pain after a fall from his truck. His past medical history revealed multiple rib fractures after a previous high energetic trauma, an emergency laparotomy after multiple gunshot wounds in the abdomen, endovascular aneurysm repair (EVAR) for a symptomatic abdominal aortic aneurysm and a recanalization of the right anterior tibial artery. After cleaning the cabin of the truck, he leaned on a broom which was standing close to him at ground level. While losing his balance, the patient fell with his right groin directly on the broom, perforating the scrotum and inguinal canal ending in the intra-abdominal cavity. On physical examination, the patient was clinically stable without signs of a major bleeding (heart rate 65, blood pressure 116/70 mm Hg). The abdomen appeared tender and a swollen right groin was noted together with a herniating right testis through the lacerated scrotum. A computed tomography (CT) thorax-abdomen showed a traumatic inguinal hernia with herniation of omentum and intestines (Fig. 1). Intra-abdominal air and fluid was seen around the herniation and perforation of intra-abdominal organs was expected.

The patient was transferred to the operation theatre for exploration by a multidisciplinary team (trauma surgeon, gastrointestinal surgeon and a urologist). An incision was made at the inguinal canal, proximal of poupart's ligament followed by opening scarpa's fascia and the aponeurosis of the external oblique muscle. The spermatic duct and testicular vessels were inspected and intact. The deep inguinal ring was lacerated. The laceration was big enough to inspect the intestines showing no signs of injury. Further exploration showed no evidence for fecal spill, perforations, contamination or abdominal bleedings besides a small hematoma. Intra-abdominal adhesions did not affect the surgical procedure. After the inguinal canal was lavaged, the abdominal wall was closed following a Lichtenstein procedure including the usage of a mesh patch [1]. After closing the abdominal wall, the testis and the scrotum were inspected. The right testis was vital, the tunica dartos was intact and only a small hematoma was seen at the testis. The lacerated scrotum was repaired. After surgery, the patient was administered intravenous amoxicillin/clavulanic acid for 4 days as prophylaxis. The recovery was delayed due to a reactive small bowel ileus. A postoperative CT scan depicted no signs of perforation or intra abdominal abscesses. After eleven days, the patient was discharged and started a rehabilitation program. At 3 months follow up no complications were reported.

## Discussion and conclusions

Inguinal hernias are among the most common abdominal wall hernias. However, inguinal hernias due to a direct trauma are very rare.

Most reported traumatic hernias are the result of blunt trauma due to motorcycle- or bike handlebars or during sports. These hernias are typically the result of a sudden increase of intra abdominal pressure disrupting the fascia and muscles. Inguinal hernias as a result of penetrating trauma are less frequent. A case published in 2013 reported a defect of the inguinal canal after perforation of a bulls horn [2]. An 8–10 cm defect was seen in the posterior wall of the inguinal canal. An open approach was used for exploration of the abdomen and viscera. The inguinal region was reconstructed in layers. Zang et al. reported the penetration of a steel bar into a patient's right groin [3]. The clinicians chose an iliofemoral approach with cutting the inguinal ligament to expose the vessels and nerves at risk, and retrieved the steel bar out of the patient. Both cases did not mention the use of a mesh patch to reinforce the abdominal wall.

Traumatic inguinal hernias can be treated differently: local exploration, a laparoscopic approach or a laparotomy. If at initial presentation a severe concomitant intra-abdominal injury is suspected, a laparotomy would be preferable. In other situations, local inspection or laparoscopy might be the optimal approach minimalizing the surgical impact [4].

The use of mesh in the acute phase of traumatic abdominal wall hernias is a matter of debate. A traumatic hernia repair at initial presentation might prevent additional surgery and avoid complications like incarcerations. However, there might be an increased risk of surgical infections [5].

In this case, the defect of the abdominal wall was big enough to locally inspect the intestines and abdominal cavity without converting to a laparotomy or laparoscopy. Since no fecal spill or blood clots were noted at the inguinal approach, we decided not to convert to a laparotomy or laparoscopy. The absence of signs of contamination resulted in the usage of a mesh patch. Watchful wait and see was initiated. No post operative infections or recurrence of herniation occurred.

We present a case of a patient with a traumatic inguinal hernia after penetrating injury in the inguinal canal, without perforation or bleeding of the small bowels, and treated safely with local inspection and direct repair using a mesh patch.

## Abbreviations

EVAR: endovascular aneurysm repair
CT: computed tomography

## Take home messages

- Traumatic inguinal hernias due to penetrating trauma are rare.
- Consider local exploration if enough exposure is reached.

## Declaration of competing interest

None.
